# Targeted matrisome analysis identifies thrombospondin-2 and tenascin-C in aligned collagen stroma from invasive breast carcinoma

**DOI:** 10.1038/s41598-018-31126-w

**Published:** 2018-08-28

**Authors:** Lucas A. Tomko, Ryan C. Hill, Alexander Barrett, Joseph M. Szulczewski, Matthew W. Conklin, Kevin W. Eliceiri, Patricia J. Keely, Kirk C. Hansen, Suzanne M. Ponik

**Affiliations:** 1 Department of Cell and Regenerative Biology, University of Wisconsin-Madison, 1111 Highland Ave., WIMR II Rm. 4528, Madison, WI 53705 United States; 2 Department of Biochemistry and Molecular Genetics, University of Colorado-Denver, 12801 E. 17th Avenue, Bldg. RC-1 South, Aurora, CO 80045 United States; 3 Laboratory for Optical and Computational Instrumentation, University of Wisconsin at Madison, 1675 Observatory Dr., Madison, WI 53706 United States

## Abstract

Increasing evidence demonstrates an important role for the extracellular matrix (ECM) in breast cancer progression. Collagen type I, a core constituent of the fibrous ECM, undergoes a significant set of changes that accompany tumor progression, termed Tumor Associated Collagen Signatures (TACS). Late stages of this progression are characterized by the presence of bundled, straight collagen (TACS-2) that become oriented perpendicular to the tumor-stromal boundary (TACS-3). Importantly, the presence of TACS-3 collagen is an independent predictor of poor patient outcome. At present, it remains unclear whether reorganization of the collagen matrix is the consequence of mechanical or compositional tissue remodeling. Here, we identify compositional changes in ECM correlating to collagen fiber reorganization from nineteen normal and invasive ductal carcinoma (IDC) patient biopsies using matrisome-targeted proteomics. Twenty-seven ECM proteins were significantly altered in IDC samples compared to normal tissue. Further, a set of nineteen matrisome proteins positively correlate and five proteins inversely correlate with IDC tissues containing straightened collagen fibers. Tenascin-C and thrombospondin-2 significantly co-localized with aligned collagen fibers in IDC tissues. This study highlights the compositional change in matrisome proteins accompanying collagen re-organization during breast cancer progression and provides candidate proteins for investigation into cellular and structural influences on collagen alignment.

## Introduction

Several hallmarks of tumor formation have been proposed and include the evasion of apoptosis, uncontrolled proliferation, self-sufficiency in growth, angiogenesis, and tissue invasion and metastasis^1^. The extracellular matrix (ECM) impinges upon the regulation of each of these hallmark processes, resulting in the loss of normal tissue architecture^2–4^. At the tissue level, tumor progression is accompanied by an increase in the deposition of collagen within the stromal ECM^5–8^. In addition to increased deposition of collagen, the architecture of the collagen stroma also greatly influences tumor progression. Distinctive patterns of collagen reorganization occur during breast cancer progression, termed Tumor-Associated Collagen Signatures (TACS)^9,10^. Briefly, the descriptions of the organization are as follows: normal - collagen appears wavy, curly, and randomly organized; TACS-1 - collagen that still appears wavy, curly, and random, but increased fiber accumulation is observed near tumor masses; TACS-2 - collagen fibers are straight and largely align parallel/tangential to the tumor boundary; TACS-3 - straight collagen fibers align perpendicular to the tumor boundary. TACS-3 was found to be a prognostic indicator of poor patient outcome^11^. It is unclear whether reorganization of collagen is the consequence of mechanical cues or aberrant ECM deposition. In combination, these studies all suggest an underappreciated role for collagen alignment in metastatic disease progression that requires additional investigation of compositional changes in the ECM that accompany collagen reorganization.

The past decade of ECM-focused mass spectrometry research has allowed for great strides to be made in both the biological characterization and technical feasibility of increasing our understanding of normal tissues and the changes that occur in diseases. The ever-increasing library of novel ECM and matricellular proteins allows for enhanced understanding of the many roles the ECM plays in development and disease. It also allows for the development of high-throughput methods that can be applied to virtually any disease and grant a more comprehensive snapshot of the underlying dysregulations^12–17^.

In this study, we identified ECM proteins that change between normal mammary tissue and invasive ductal carcinoma (IDC) with respect to collagen fiber organization. In human patient samples, normal biopsy tissue possessed curly, randomly organized collagen fibers. The IDC tissues, however, could be further categorized into ‘curly’ and ‘straight’ (IDC-c and IDC-s) sub-categories, allowing for investigation into proteins associated with collagen alignment. We identified nineteen ECM proteins that positively correlate and five ECM proteins that inversely correlate with aligned collagen fibers in IDC-s tissues. These proteins were further characterized for relevance to disease outcome, structural localization, and association with aligned collagen fibers. Based on these parameters, a signature of four, IDC-s associated proteins that predict metastatic outcome were identified. Two proteins from this signature, tenascin-C and thrombospondin-2, co-localize with aligned collagen fibers in IDC-s patient samples. Overall, this study provides a set of candidate ECM proteins for further investigation into the mechanisms that may facilitate collagen fiber organization during tumor progression.

## Results

### Targeted matrisome proteomics reveals a unique signature of ECM and cellular proteins in invasive ductal carcinoma tissues compared to normal breast tissues

**Figure 1:**
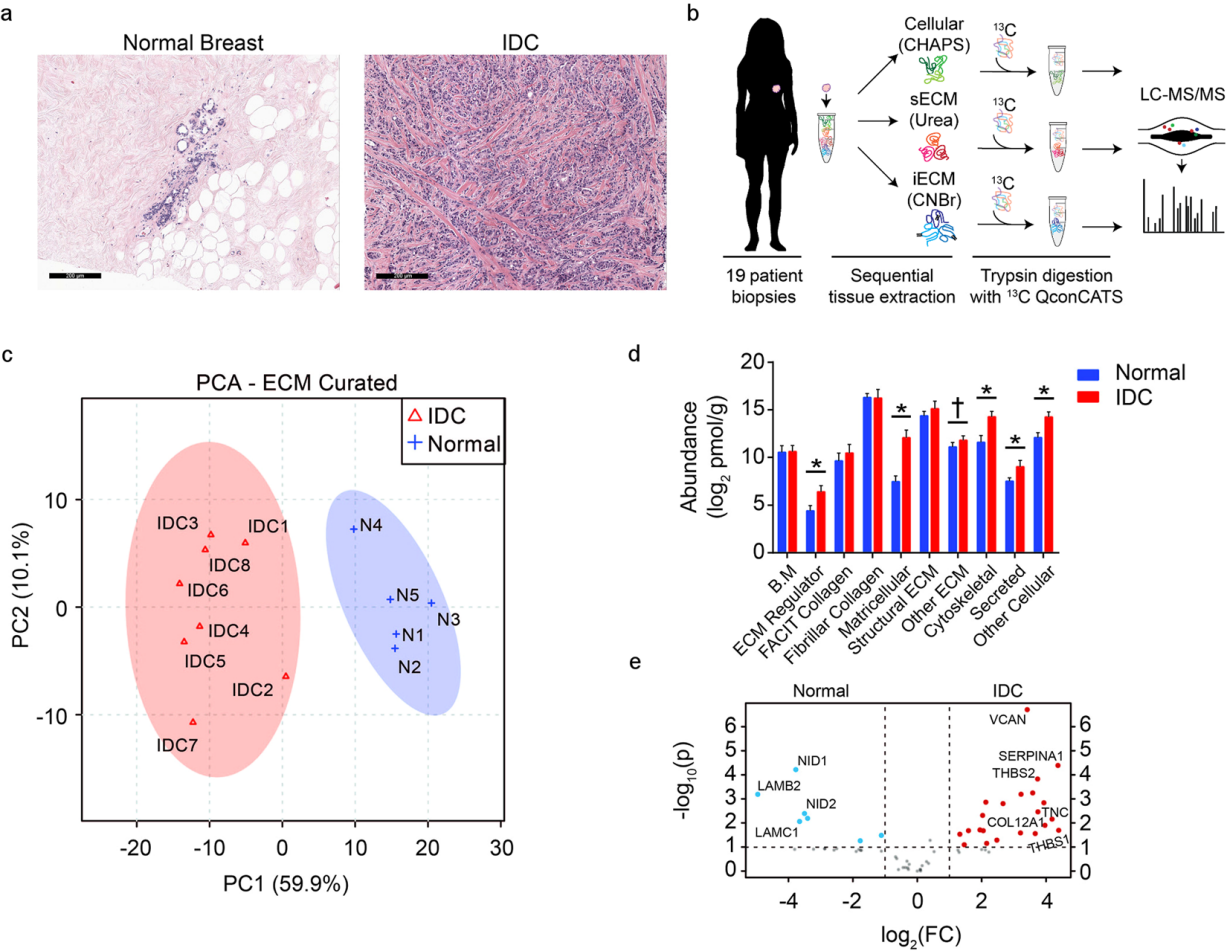
Targeted extracellular matrix proteomics reveals compositional changes between normal breast and IDC patient samples. (**a**) Representative H&E images of normal breast and invasive ductal carcinoma (IDC) histological sections. (**b**) Cartoon depicting the workflow for processing the frozen, human, biopsy cores, highlighting the cellular (CHAPS), soluble ECM (urea, sECM), and cyanogen bromide, insoluble ECM (CnBr, iECM) fractions from each sample before LC-MS/MS analysis. (female silhouette obtained from https://www.clipartqueen.com) (**c**) Principle Component Analysis of the quantitative ECM profile between normal breast (n = 5) and IDC (n = 8) conditions. (**d**) Bar graph representing the quantitative ECM results between normal breast and IDC samples, grouped by gene ontology, functional classifications. Error bars represent standard deviation to the mean. *p < 0.01, ^†^p = 0.06, WRS. (**e**) Volcano plot comparing the fold change of ECM proteins between IDC and normal breast. Significance threshold set to p < 0.01, Student’s t-test.

In healthy tissue, H&E staining reveals well-ordered organization of the normal breast architecture. The breast lobules and ducts are immediately surrounded by basement membrane and circumscribed by collagen-rich stroma and adipose tissue. In the IDC-diagnosed patient samples, the epithelial organization has been lost and not only has the surrounding stroma changed in appearance, but it has also become more intercalated and woven throughout the cellular mass (Fig. 1a). To determine the composition changes within the ECM of each patient cohort, a LC-MS/MS analysis was completed. To mass-balance total ECM protein abundance among the sequential fractions each patient sample was processed by three, different serial extractions (cell-associated, CHAPS; soluble ECM, urea; and insoluble ECM, CnBr), followed by inclusion of stable-isotope labeled peptide standards targeting ECM and ECM-associated proteins (Fig. 1b). LC-SRM analysis of these samples resulted in the absolute quantification of ninety-four ECM, ECM-associated, and cellular markers of interest (Supplementary Table 1). Based on this quantitative profile of proteins, a principal components analysis was carried out and revealed two distinct proteomic profiles corresponding to the IDC and normal breast sample populations (Fig. 1c).

ECM-targeted proteomics identified significant changes in protein abundance between the IDC and normal tissues (Fig. 1d,e). Among the most significantly down-regulated proteins in the IDC tissues are several basement membrane proteins, laminin β2 (LAMB2), laminin γ1 (LAMC1), and nidogens -1 and -2 (NID1/NID2). This finding is in line with the known loss of the basement membrane during tumor progression. Interestingly, several of the most significantly up-regulated ECM proteins in the IDC tissues, including versican (VCAN) and tenascin-C (TNC), are biomarkers for breast cancer^18–21^. The serine protease inhibitor, serpin A1 (SERPINA1), along with THBS-1 and THBS-2 were also among the other abundant proteins with highly significant changes compared to the normal tissues. The only significantly changed collagen family member in this analysis was a non-fibrillar, FACIT (Fibril-Associated Collagens with Interrupted Triple helices) collagen, type α1 XII (COL12A1). These proteomic profiles were further classified into ten, functional, gene ontology groups to evaluate patterns of protein regulation in IDC tissue compared to normal tissue. Significant increases were found in ECM regulator, matricellular, cytoskeletal, secreted, and other cellular groups in the IDC tissue samples compared to normal tissue. In these groups several noteworthy changes include the ECM cross-linking proteins, lysyl oxidase, lysyl oxidase-like 1, and tissue transglutaminase II (TGM2), which have been previously shown to play a role in tumor stiffness and progression^22–24^. Two cytoskeletal related proteins, vimentin and plectin, which are known tumor biomarkers, were also upregulated in IDC samples compared to normal tissue^25,26^. In all, a summary of the twenty-seven, statistically significant, and trending, increased- and decreased-abundance proteins between IDC and normal breast tissues has been compiled and serves as a list of possible candidates for further investigation (Table 1).

**Table 1:** List of twenty-seven significantly changed, and trending, ECM proteins between normal breast and IDC-s samples with aligned collagen.

IDC Proteins Changed from Normal			
Protein Name	Gene Symbol	IDC/Normal (Avg Fold Δ)	Combined p-value
Laminin β2	LAMB2	0.11^‡^	0.03
Mimecan/Osteoglycin	OGN	0.36	0.03
Fibromodulin	FMOD	1.6	0.10
Biglycan	BGN	2.6	0.06
Fibulin-4	EFEMP2	3.5	0.02
TGF-β Induced	TGFBI	3.8	0.06
Aggrecan	ACAN	4.0	0.03
Fibulin-3	EFEMP1	5.0	0.02
SPARC	SPARC	5.5	0.01
EMILIN-1	EMILIN1	6.1	0.01
Lysyl Oxidase-like 1	LOXL1	7.8	0.03
Latent TGF-β Binding Protein 2	LTBP2	8.1	0.01
Periostin	POSTN	15.5	0.02
Latent TGF-β Binding Protein 1	LTBP1	28.1	0.009
Fibronectin	FN1	35.8	0.02
Collagen (α1) XII	COL12A1	39.4	0.01
Tenascin-C	TNC	64.1	0.01
Transglutaminase II	TGM2	6.9^‡^	0.03
Fibulin-1	FBLN1	8.5^‡^	0.01
Versican	VCAN	12.3^‡^	0.01
Vitronectin	VTN	15.2^‡^	0.01
Thrombospondin-1	THBS1	68.7^‡^	0.01
Aggrin	AGRN	†	0.04
Fibrillin-2	FBN2	†	0.005
Lysyl Oxidase	LOX	†	0.04
Fibulin-2	FBLN2	**	0.005
Thrombospondin-2	THBS2	**	0.005

Twenty-seven significantly changed ECM proteins between normal breast and IDC tissues. The upper two rows indicate the consistently down-regulated proteins in IDC compared to the normal tissues. The other rows indicate consistently up-related proteins in IDC compared to the normal tissues. ^‡^Protein recovered in both proteomic experiments (infinite relative fold change due to non-detectable recovery in normal tissue of one of the experiments). ^†^Protein recovered only in the targeted proteomic analysis (infinite relative fold change due to non-detectable recover in normal tissue. **Protein recovered in both proteomic experiments (infinite relative fold change due to non-detectable recovery in normal tissue samples for both experiments).

### Aligned collagen stroma in IDC samples have an altered ECM composition

**Figure 2:**
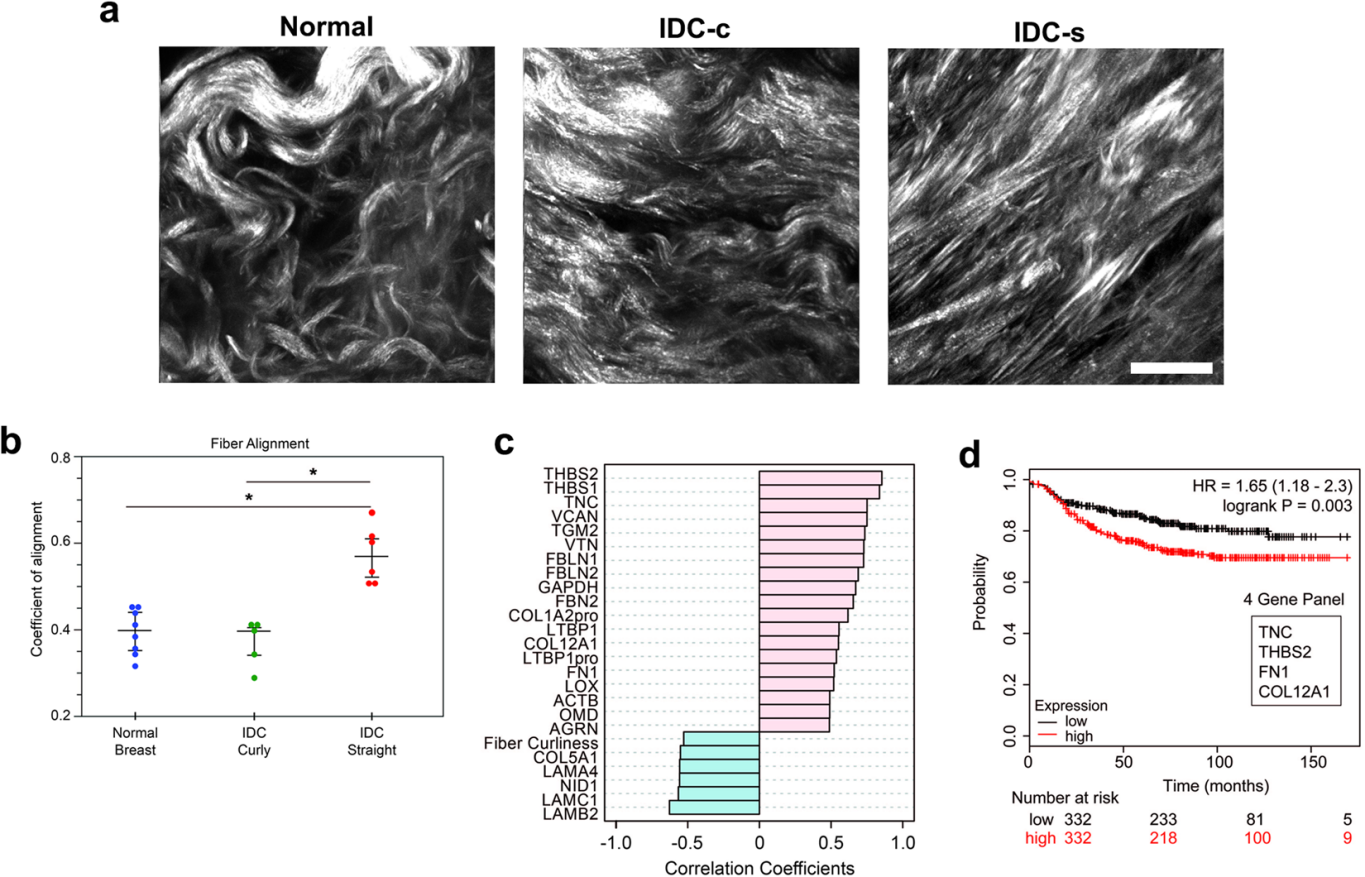
Collagen fiber orientation analysis identifies ECM proteins that correlate with collagen fiber alignment, not simply progression to IDC. (**a**) Representative second-harmonic generation (SHG) images of normal breast and IDC tissues. The normal breast tissues always exhibited curly/wavy, randomly organized collagen (left), whereas the IDC samples showed more variability. Some of the IDC samples contained the randomly organized collagen similar to normal breast (middle), while others possessed straightened/aligned collagen architectures, similar to TACS-3 (right). Images presented here are compressed z-series, ‘max intensity.’ Scale bar = 25 µm. (**b**) Box plots of ct-FIRE and CurveAlign analysis for all SHG images of patient samples used in this study. Coefficient of alignment ranges from completely random fiber orientation, 0.0, to completely aligned fiber orientation, 1.0. (**c**) Pattern Analysis of log_2_-normalized, quantitative and global proteomics results to fiber alignment (normal breast, n = 8; IDC, n = 11). (**d**) Four increased ECM proteins for IDC/normal breast (collagen XII, COL12A1; fibronectin, FN; tenascin-C, TNC; and thrombospondin-2, THBS-2) formed a heightened correlative signature for poor distant metastasis-free survival *in silico*, using the K-M plotter database, than any ECM protein individually had correlated.

To identify changes in ECM composition that accompany collagen organization, patient samples were further classified by overall collagen alignment score and correlated to the proteomic findings (Fig. 2a,b). As expected, all normal breast samples showed a relatively low alignment coefficient for their collagen organization. A subset of the IDC samples (six of eleven samples) had an increased alignment of their collagen fibers. The remaining five IDC samples had low alignment scores similar to normal tissue. Based on the coefficient of collagen alignment, IDC tissues with a low coefficient of alignment (<0.5) were classified as IDC-curly (IDC-c) and those with a coefficient of alignment >0.5 were classified as IDC-straight (IDC-s) for further analysis of ECM composition (Fig. 2b). A robust set of correlations between the proteomic results and alignment analysis identified nineteen matrisome proteins that positively correlated with collagen fiber alignment and five proteins that inversely correlate to fiber alignment. This further implied a unique ECM composition within the aligned IDC stroma (Supplementary Fig. S1). Among the highest correlations with collagen fiber alignment were the matricellular proteins TNC, THBS-1, and THBS-2, followed by VCAN and the cross-linking protein, TGM2 (Fig. 2c). Collagen (α1) V (COL5A1) and several laminin family members inversely correlated with the collagen fiber alignment. To focus our study on the importance of compositional changes relevant to patient outcome, we evaluated each of the ECM proteins that positively correlated with aligned collagen for a correlation with patient metastatic outcome. Our hypothesis was that these alignment-associated, ECM proteins would correlate with a worse metastatic outcome in patients, mimicking the TACS-3 metastatic patient correlation with increased metastases^11^. *In silico*, four ECM proteins, COL12A1, fibronectin (FN), TNC, and THBS-2, correlated with a worse distant metastasis-free survival (DMFS) patient outcome. These four genes were found to form an even stronger *in silico* correlation when combined as a signature than when evaluated individually (p = 0.003, Fig. 2d, Supplementary Fig. S2).

### IDC ECM proteins localize to the stroma compared to normal breast tissue

**Figure 3:**
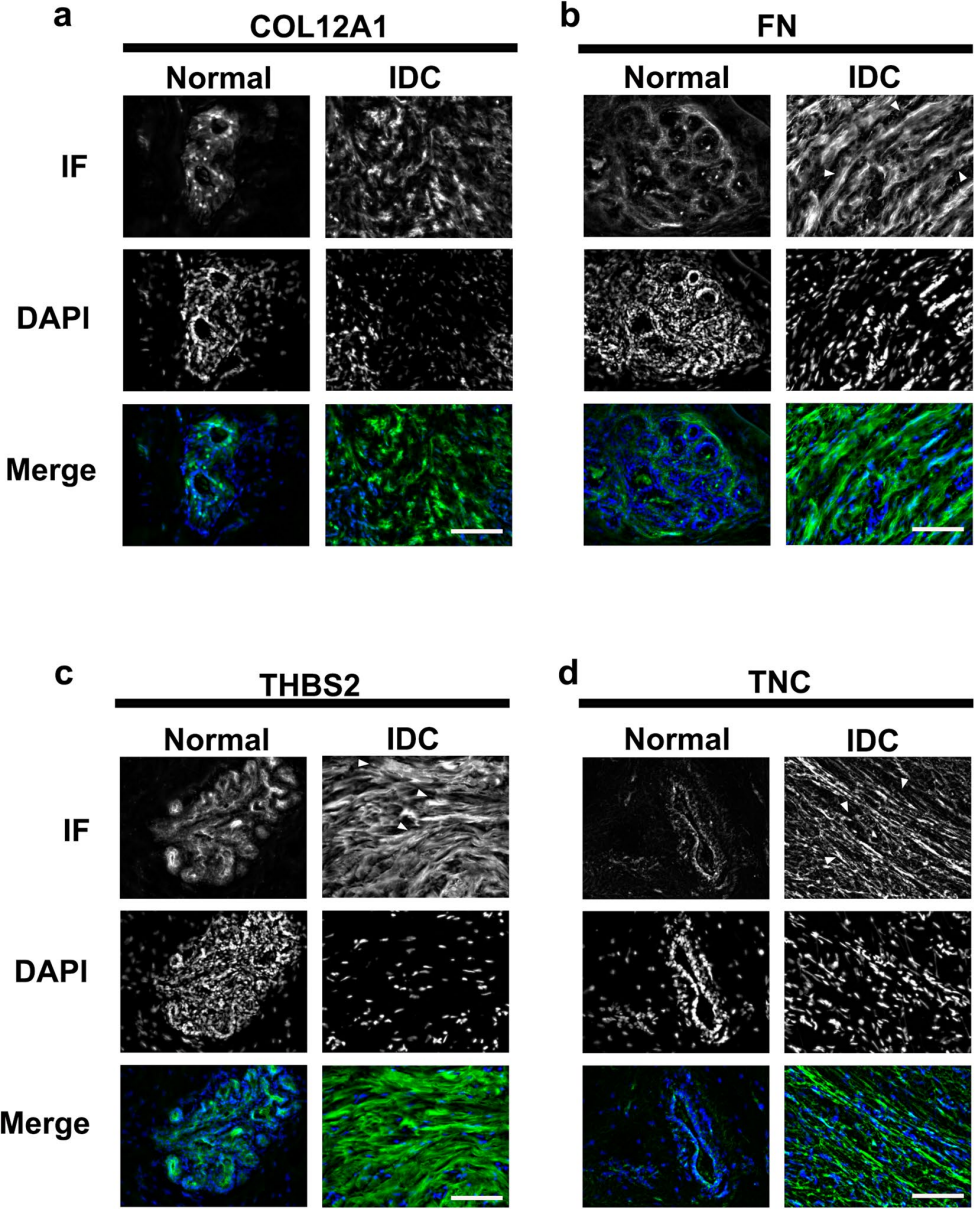
Immunofluorescence validation of the four ECM proteins in the normal breast and IDC-s patient tissues. Immunofluorescence of normal breast and IDC-s patient tissues. Representative, immunostained images of (**a**) collagen XII, (**b**) fibronectin, (**c**) thombospondin-2, and (**d**) tenascin-C. These four ECM proteins were found localized near cellular (DAPI positive) regions in the normal breast tissues. In the IDC-s tissues, the four signature proteins associated with stromal/fibrous structures. Scale bar = 25 µm. Normal breast, n = 8; IDC, n = 6.

**Figure 4:**
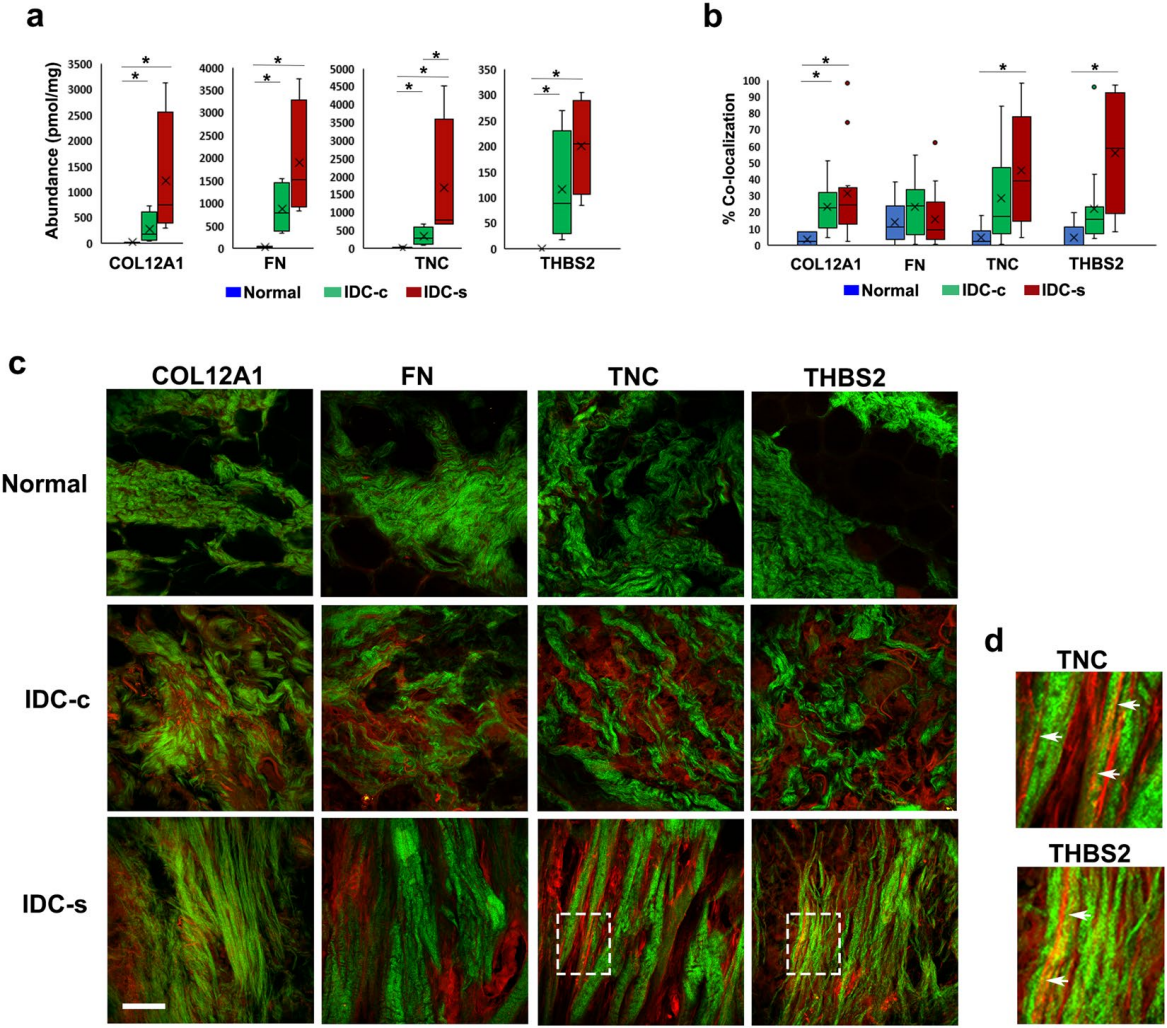
Co-localization of aligned collagen fibers with THBS-2 and TNC in normal breast and IDC-s patient tissues. (**a**) Concentrations from quantitative LC-MS/MS analysis for collagen XII, fibronectin, thrombospondin-2, and tenascin-C. All four proteins are increased in abundance in IDC-c and IDC-s compared to normal tissue. Error bars represent sample variance, X represents the mean concentration. *p ≤ 0.05. Normal breast, n = 5; IDC-c and IDC-s, n = 4. Representative, single-plane images of collagen SHG and immunostained ECM proteins in different portions of the same patients’ tissues used for LC-MS/MS. (**b**) Quantifications of percent-area fluorescence within the masked-area of collagen SHG. A minimum of six ROI’s from each patient sample were used for co-localization analysis. * Indicates p < 0.05. (**c**) Multiphoton images of COL12A1, FN, TNC, and THBS-2 (red) with collagen SHG (green) from normal, IDC-c and IDC-s patient samples. COL12A1 significantly co-localized with IDC-c and IDC-s collagen, however it did not appear organize specifically along collagen fibers. Tenascin-C, TNC shows an increase in co-localization with only with collagen fibers in IDC-s tissues. Similarly, thrombospondin-2, THBS-2, staining is co-localized with fibrillar collagen in IDC-s samples. Scale bar = 50 µm. (**d**) Magnified Insets (white boxes on IDC-s TNC and THBS2 images from panel c) of straight collagen fiber (green) co-localization with TNC or THBS2 (red). Arrowheads indicate regions of co-aligned collagen and ECM protein IF.

Focusing on the four-matrix protein signature correlated with patient outcome, we determined the localization of the ECM proteins in IDC and normal tissue (Fig. 3). In normal breast tissue, all four matrix proteins, COL12A1, THBS-2, FN and TNC, are low in expression but were still detectable by immunofluorescent analysis. The ECM proteins localized near the DAPI positive, cellular structures in normal breast tissue (Fig. 3a–d). When observing the staining in the IDC tissues, COL12A1, FN, TNC and THBS-2 were found localize within the disorganized stromal compartment. (Figure 3a–d). The change in spatial organization of ECM proteins in IDC tissue is consistent with the overall increase in abundance of the signature matrix proteins (Fig. 4a) and loss of epithelial architecture compared to normal biopsy tissue.

### THBS-2 and TNC show an increased co-localization with collagen fibers in IDC-s samples

To identify candidate mechanisms of collagen fiber alignment, each ECM protein in the four-protein signature was examined for localization in the stomal of IDC-s, IDC-c, and normal breast tissue samples. Quantitative proteomic analysis revealed an increase in all four signature proteins, starting from low or absent concentrations in normal tissue biopsies, elevated concentration in IDC-c tissue and significantly enhanced concentrations in the IDC-s tissue (p = 0.05, (WRS) Fig. 4a). Next, the increase in abundance of these four proteins was assessed for localization with collagen SHG by multiphoton imaging (Fig. 4b,c). Approximately 20–30% of COL12A1 co-localized with SHG in both IDC-c and IDC-s tissue samples (p = 0.05, (WRS) Fig. 4b). The immunofluorescent signal for COL12A1 was high across the whole stromal region and was not specifically enhanced near or along collagen fibers. Fibronectin was identified in the stromal compartment of IDC tissue but did not co-localize with collagen SHG in either IDC-c or IDC-s tissue. Importantly, THBS-2 and TNC significantly co-localized with collagen fibers in the IDC-s tissue but did not co-localize with collagen in the IDC-c samples (p = 0.05, and p = 0.01, (WRS) respectively; Fig. 4b–d). Further, both THBS-2 and TNC form fiber structures that localize directly along straight collagen fibers (Fig. 4d) Since both TNC and THBS-2 co-localize with collagen fibers, these two proteins may be important candidates for future investigation into the mechanisms of collagen fiber reorganization during tumor progression.

## Discussion

We have previously described a set of collagen architectural changes (TACS-3) in the tumor microenvironment that correlates with poor outcome in breast cancer patients^11^. The potential of this ECM biomarker indicates a need to better understand the process of collagen reorganization during tumor progression. In particular, we sought to determine whether collagen reorganization was accompanied by changes in the composition of the ECM. Here, we use a robust and quantitative technology to determine that twenty-seven ECM proteins change in abundance between normal breast and aligned IDC stroma. The proteins upregulated in IDC samples span several ontological categories. Not surprising, this suggests that disease progression involves both cellular and structural matrix proteins that may play a role in mechanical or signaling properties of the fiber network. Further characterization of IDC patient samples by collagen fiber organization enabled the specific identification of an ECM profile that correlates with aligned collagen architecture. Using the collagen fiber alignment coefficient, a set of twenty-four matrix proteins was identified and four of these alignment-associated proteins formed a correlative signature with distant metastasis. The localization of the four-protein signature, COL12A1, FN, THBS-2, and TNC, revealed that only the matricellular proteins, THBS-2 and TNC, have increased co-localization with aligned collagen fibers in IDC-s tissue. However, it is well known that FN generates a provisional template that directs the assembly of more permanent collagen fibers^27–29^. Likewise, a COL12A1 binds directly to collagen type I and is thought to regulate organization and mechanical properties of collagen fibril bundles^30^. These findings demonstrate the complex role of matrisome proteins in collagen reorganization in the breast tumor microenvironment.

Thrombospondins are a family of large (~450 kDa, trimeric) matricellular proteins that can dynamically interact with both cells and structural ECM proteins^31,32^. They have previously been examined in non-malignant and IDC mammary tissues^33,34^. Similar to our findings, thrombospondin has been shown to localize near normal epithelial structure and thrombospondin was elevated within IDC tissue due to active secretion by carcinoma cells. Additionally, it is know that THBS-1 and -2 have a binding region for collagens type I–V, allowing a direct interaction between THBS and collagen^35^. In the context of breast cancer, blocking cellular interactions with THBS-1 and -2 is sufficient to set off a “stromal program” of increased ECM deposition in mammary tissue^36^. Another investigation of breast cancer determined that inhibiting tumor cell interactions with THBS-2 reduced tumor growth and metastasis to lymph nodes and the lungs, further supporting an important role for THBS-2 in breast cancer progression^37^.

TNC is the other matricellular ECM protein that was found in high association with aligned collagen fibers. TNC has long been known to correlate with worse metastatic and disease outcomes in patients, which parallels the correlation of worse outcome by TACS-3^38–40^. Further, TNC has recently been identified as a potential cancer-associated fibroblast marker for breast ductal carcinoma^41^. TNC was first described as a modulator of cell adhesion, but it also directs cell signaling and gene expression programs by shaping mechanical and biochemical cues within the cellular microenvironment. Recently, TNC was shown to activate epithelial to mesenchymal transition in breast cancer cell lines, further suggesting it has a strong influence on cell signaling during tumor progression^42^. TNC has multiple ECM binding partners, the best characterized being the interaction with FN. FN has an important role as a ‘master matrix assembly’ regulator, with binding sites for many ECM proteins, glycoproteins, and serves as a platform for collagen fibrillogenesis^28,43^. TNC also binds collagens, periostin, and fibrillin-2, however the binding site of these interaction has not yet been mapped^39^. The role of TNC in inter-matrix interactions suggests a structural role potentially involved in the stiffness of the ECM, but the interactions could feasibly also modulate how the ECM components signal to cells.

In summary, this study utilized matrisome-targeted proteomics to highlight the compositional changes of the ECM accompanying collagen fiber alignment in a specific subset of IDC tissues. The targeted proteomic approach presented here also provides novel candidate matricellular, and related, proteins that may play an important role not only in cancer, but also other chronic diseases involving ECM architectural changes and their progression^44^. The two key proteins identified in this analysis, TNC and THBS-2, have both cell signaling and structural functions in the tumor microenvironment; thus, providing insight into the complex mechanisms of why collagen signatures have prognostic significance. Our results suggest structural function for these two proteins based on their co-localization with aligned collagen, however further investigation will be necessary to determine their exact role in collagen fiber alignment during tumor progression. In the future, this ECM profile may serve as a useful biomarker to enhance the early diagnosis of patient biopsies for the risk of developing distant metastases or as a suite of possible targets for therapeutic intervention to disrupt disease progression.

## Methods

### Tissue Acquisition

De-identified, human breast sample cores were obtained from the UW Carbone Cancer Center (UWCCC) Translational Science Biocore (TSB) Biobank under the Minimal Risk IRB (MR12 IRB, 2016-0934-CR001). All experiments were approved by the MR-IRB as part of the application process for acquiring UWCCC TSB biobanked human tissues. Informed consent was obtained upstream of research by UWCCC TSB staff, or affiliates, from all participants and/or their legal guardians. All research was conducted according to the MR-IRB guidelines and regulations, as outlined in the University of Wisconsin Knowledgebase:

[The MR-IRB reviews and approved research accordance with the laws of the United States of America and the State of Wisconsin. The MR-IRB complies with the applicable requirements of the Department of Health and Human Services (DHHS) regulations, 45 CFR Part 46; the Food and Drug Administration (FDA regulations, 21 CFR Parts 50, 56, 312, and 812; Veteran’s Administration (VA) Regulations pertaining to the protection of human subjects, 38 CFR Part 16; and the privacy requirements of the Health Insurance Portability and Accountability Act of 1996 implemented by 45 CFR Parts 160 and 164 (Privacy Rule). The University of Wisconsin - Madison holds a current Federalwide Assurance (FWA) from the Office of Human Research Protection (OHRP) at the United States Department of Health and Human Services, #FWA00005399, which is periodically updated as required by OHRP].

Requests were made for normal mammary tissue and invasive ductal carcinoma (IDC) diagnosed mammary tissue that consist of at least 70% cellularity. The tissues were received as flash-frozen biopsy cores. A total of eight normal breast tissue samples and eleven IDC tissue samples were used in these experiments.

### Tissue Fixation and Preparation for Two-Photon Microscopy Imaging

From each frozen sample obtained from the TSB biobank, a small portion was formalin fixed (Protocol, cat. # 264–584) for 48 hrs. The remainder of the uncut frozen tissue was prepared for proteomic analysis. Formalin-fixed sample portions were embedded in 5% agarose and sectioned by vibratome. A 200–300 mm section from the middle-portion of each tissue fragment was selected for imaging. (Leica Vibratome 1200 S; Leica Biosystems, Buffalo Grove, IL).

### Tissue Preparation and Extractions

For the global proteomics, flash frozen samples were thawed at room temperature and minced finely before exposure to freshly-made, 8 M urea solution. Samples were incubated with constant rotation for 1 week at room temperature. The mixture was then centrifuged at 14k RPM for 10 min. and the supernatant of urea extract was collected into a fresh tube. To verify ECM extraction, samples were dialyzed into 1.8 M urea over two days, and then dialyzed into 50 mM Tris-buffer, pH 7.4. Fibronectin and pan-tenascin were analyzed via western blotting (data not shown) to determine extraction efficiency.

For targeted, quantitative proteomics, tissues were prepared as previously described^45,46^. Briefly, flash frozen tissues were pulverized using a mortar and pestle under liquid nitrogen conditions. All samples were measured to ~10 mg and underwent sequential extractions with CHAPS buffer, 8 M urea, and then cyanogen bromide (CnBr) digestion. All three fractions were quantified for protein content using the Pierce 660 nm Protein Assay Reagent (cat. no. 22660). Prior to enzymatic digestion, samples were spiked with a stable-isotope labeled library of concatemerized peptides representing ECM proteins of interest^47,48^.

### Liquid Chromatography - Mass Spectrometry and Data Analysis

Samples were analyzed by both liquid chromatography – selected reaction monitoring (LC-SRM) and liquid chromatography – data dependent acquisition (DDA) tandem mass spectrometry (LC-MS/MS) as previously described^46,48^. Global analysis was performed on an Orbitrap – Velos coupled with an Eksigent 2D nano-LC, while targeted (LC-SRM) analysis was performed on a Qtrap 5500 coupled with a Dionex Ultimate 3000 UHPLC utilizing optimized conditions described previously. LC-SRM data was directly loaded into Skyline and transition quality, peak shape, and peak boundaries were manually validated. Resulting integrated peak areas were directly exported and protein quantity (pmol/g) was calculated against the known spike of stable isotope labeled peptides. LC-MS/MS data was queried against the SwissProt human database using Mascot (v2.3.1) and directly loaded into Scaffold™ (Proteome Software). Peptide Spectral Matches (PSMs) were directly exported with a 99% confidence in protein identifications and at least 2 unique peptides per protein, resulting in a false discovery rate of 0.54%. Statistical analysis for proteomics data, including principal component analysis, was performed using the MetaboAnalyst (v3.0) software suite^49^.

### Two-photon Microscopy (TPM), Second-harmonic Generation (SHG), and Two-photon Fluorescence Imaging

Collagen has a unique property, based on its chemistry and triple-helical structure, that allows for label-free imaging using second-harmonic generation (SHG) contrast on a multiphoton microscope^50–52^. All samples were imaged on custom-built, multiphoton microscopes at the Laboratory for Optical and Computational Instrumentation (LOCI, UW-Madison) consisting of a Nikon TE300 inverted microscope inverted, Spectra Physics Mai Tai DeepSee or Coherent Chameleon XR laser path, and Hamamatsu H7422P-40 photomultiplier. SHG was collected using a laser excitation of 890 nm with emission filtration using a Semrock Brightline® 445/20 nm filter. Alexa-488 labeled antibodies were excited at 890 nm with emission filtration using a Semrock Brightline® 520/35 nm filter. Alexa-594 labeled antibodies were excited at 780 nm with emission filtration using a Semrock Brightline® 615/20 nm filter. For thick tissue, vibratome sections were placed on #1.5 glass-bottom dishes (MatTek, part no. P50G-1.5-30-F) and two or three randomly chosen locations in each of the tissues were imaged as a z-series that encompassed ~100 µm depth at 10 µm step-size using a Nikon CFI Apo λS 40X WI objective. For slides, a minimum of 6 images from each tissue section were collected as a single plane capture at 1024 × 1024 pixels using a Nikon S Fluor 40x oil objective lens. Images were processed using FIJI software^53^.

### ct-FIRE and CurveAlign Collagen Architecture Assessment

Collagen fibers were identified from SHG images of patient tissues using the ct-FIRE software package (loci.wisc.edu/software/ctFIRE, v.2.0b). Further analysis of fiber to fiber orientation was completed using the CurveAlign software package (loci.wisc.edu/software/curvealign, v.4.0b). Specifically, the analysis parameters included: ‘CT-FIRE Fibers’ analysis method and set to ‘No Boundary’ boundary method; ‘Minimum nearest fibers’ to 8; and ‘minimum box size’ to 64. Finally, post-processing in CurveAlign was carried out on the ‘CA Out’ folder results, selecting the ‘combine feature files,’ to generate the final fiber orientation assessment for all patient sample images.

### KM-Plotter Database

Analysis of the impact of significantly-changed proteins between normal breast and IDC-s ECM proteins was carried out using the KM-Plotter web tool (kmplot.com) for breast cancer patients^54^. Briefly, this database was constructed using data from cancer patients whose outcome is known and who also had mRNA microarray analysis performed on their whole tumors. Each of the significantly-changed proteins from the LC/MS-MS analysis were queried against the distant metastasis-free survival (DMFS) database, n = 1,610, with the population split by the median expression values. DMFS queries had no reason to be restricted over a specific follow-up timeline and were left ‘open’ in the software settings. The results reported here were generated using the ‘2014’ database version. The statistical significance cutoff was set at p = 0.05.

### Immunofluorescence Staining

A section of the same patient samples used for LC-MS/MS were obtained as OCT-embedded blocks and sectioned at 10–20 µm thickness. Briefly, slides were fixed in cold acetone, rinsed in cold TBS and blocked with10% BSA in TBS. Primary antibody incubations were carried out in 1% BSA overnight at 4 °C. Antibodies and concentrations used were: anti-collagen XII 1:500 (Thermo, PA5-38890); anti-fibronectin 1:1,000 (BD Biologicals, cat. no. 610077); anti-tenascin-C 1:500 (Abcam, 108930); anti-thrombospondin-2 1:500 (Novus Biologicals, cat. no. 24420002). Secondary antibody incubations were performed using 1:500 dilutions in 5% BSA (TBS) for 1 hr. at RT using Alexa-488 or Alexa-594 (Thermo-Fisher cat. no. A-11034, A-21206). Nuclear counter-staining was performed with 1:10,000 DAPI before mounting with compatible aqueous media (CC Mount, Sigma-Aldrich, product no. C9368).

### Epi-fluorescence Image Collection

Initial immunostaining results were collected using a Nikon Eclipse TE300 inverted microscope with a Nikon Plan Fluor ELWD 20X/0.45 DIC L objective lens and an ORCA-R2 Digital CCD camera (part no. C10600-10B). A mercury arc lamp was used for excitation in conjunction with a Semrock “Pinkel” excitation filter set (part no. DA/FI/TX-3 × -A). Images were acquired using SlideBook Software (v5.0.034) and processed using FIJI software.

### Co-Localization, Percent Area, Calculation

A minimum of six ROIs from each patient sample were examined for co-localization with collagen fibers. For each ROI captured, the SHG and fluorescent images were thresholded to identify the % area for each. A binary mask of the SHG thresholded image was multiplied to the binary masked ECM fluorescent image to identify co-localized pixels. Using the measure tool in FIJI, the total area of co-localized pixels were divided by total area of ECM fluorescent pixels to determine the % co-localization. Co-localization of each ECM protein with collagen was determined for normal, IDC-s and IDC-s tissue.

### Statistical Analyses

Statistics were performed using MSTAT, version 6.3.1 (developed by Norman Drinkwater, McArdle Laboratories, UW-Madison). All comparisons were carried out by using the Wilcoxon rank sum test (WRS), significance p ≤ 0.05. Fisher’s method for combining p-values was used to combine the statistical results between the two mass spectrometry experiments. Dot plots represent the mean and middle two quartiles of each sample distribution.

## Electronic supplementary material

Supplemental figures

Supplemental MS Dataset 1
