# Supplementary material for: Targeted matrisome analysis identifies thrombospondin-2 and tenascin-C in aligned collagen stroma from invasive breast carcinoma
Source: Sci Rep. 2018 Aug 28;8:12941. doi: 10.1038/s41598-018-31126-w (PMC6113240; doi:10.1038/s41598-018-31126-w)
Supplement: Supplementary file 1 — Supplemental figures [file 41598_2018_31126_MOESM1_ESM.pdf]

Targeted matrisome analysis identifies thrombospondin-2 and tenascin-C in aligned collagen stroma from invasive breast carcinoma

Lucas A. Tomko[1], Ryan C. Hill[2], Alexander Barrett[2], Joseph M. Szulczewski[1], Matthew W. Conklin[1], Kevin W. Eliceiri[3], Patricia J. Keely[1], *Kirk C. Hansen[2], *Suzanne M. Ponik[1]

[1]Department of Cell and Regenerative Biology, University of Wisconsin-Madison. 1111 Highland Ave., WIMR II Rm. 4528, Madison, WI 53705

[2]Department of Biochemistry and Molecular Genetics, University of Colorado-Denver. 12801 E. 17th Avenue, Bldg. RC-1 South, Aurora, CO, 80045

[3]Laboratory for Optical and Computational Instrumentation, University of Wisconsin at Madison. 1675 Observatory Dr., Madison, WI 53706

***Co-Corresponding authors:**

Kirk Hansen, tel. (303) 724-5544, e-mail Kirk.Hansen@UCDenver.edu

Suzanne Ponik, tel. (608) 265-5094, e-mail ponik@wisc.edu

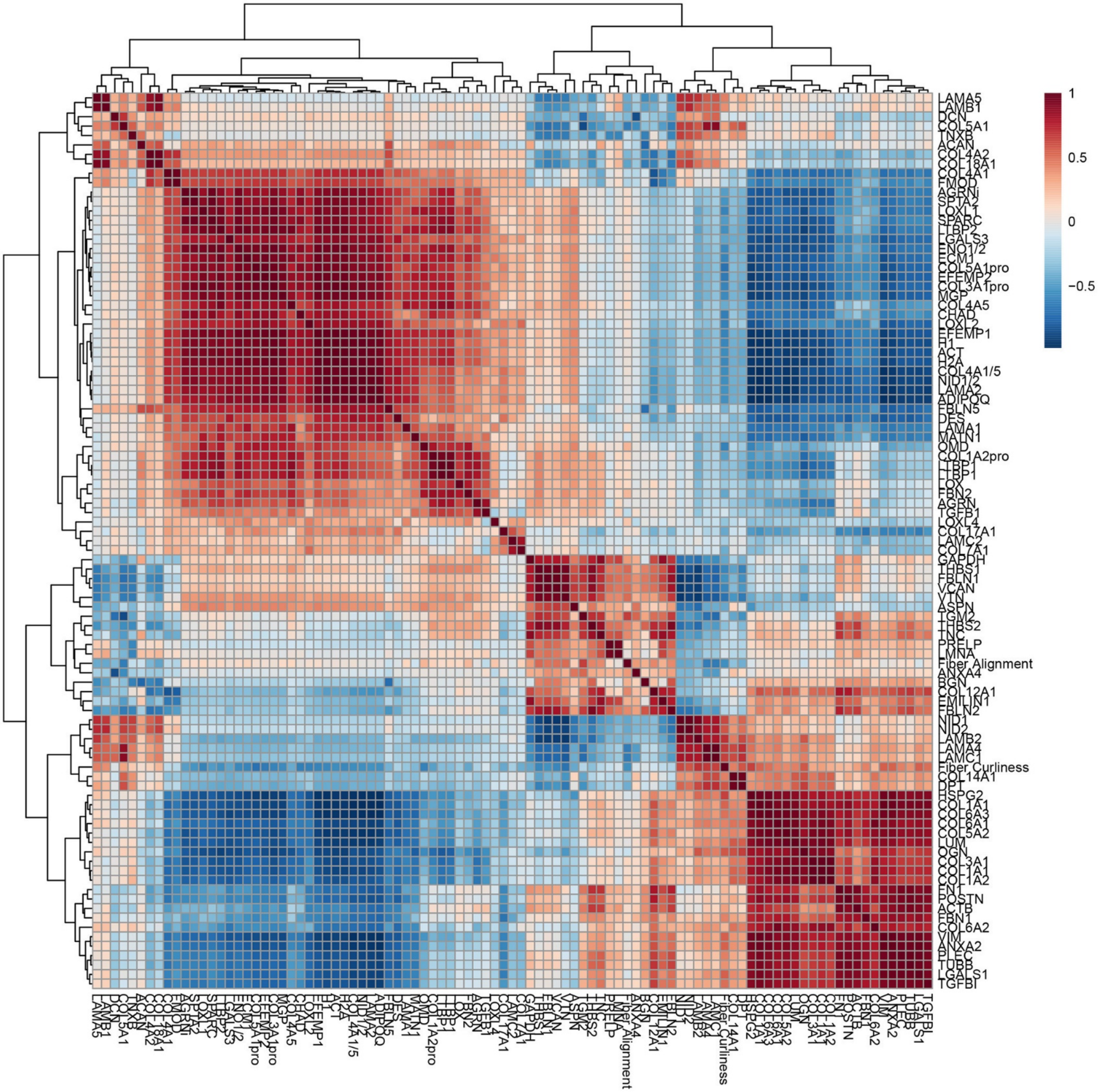

Supplementary Fig. S1. *Heat map of correlations within the global and targeted proteomics datasets.* Correlation Analysis of log2-normalized, quantitative and global LC-MS data, including fiber curliness and fiber alignment metrics from ct-FIRE/CurveAlign analysis.

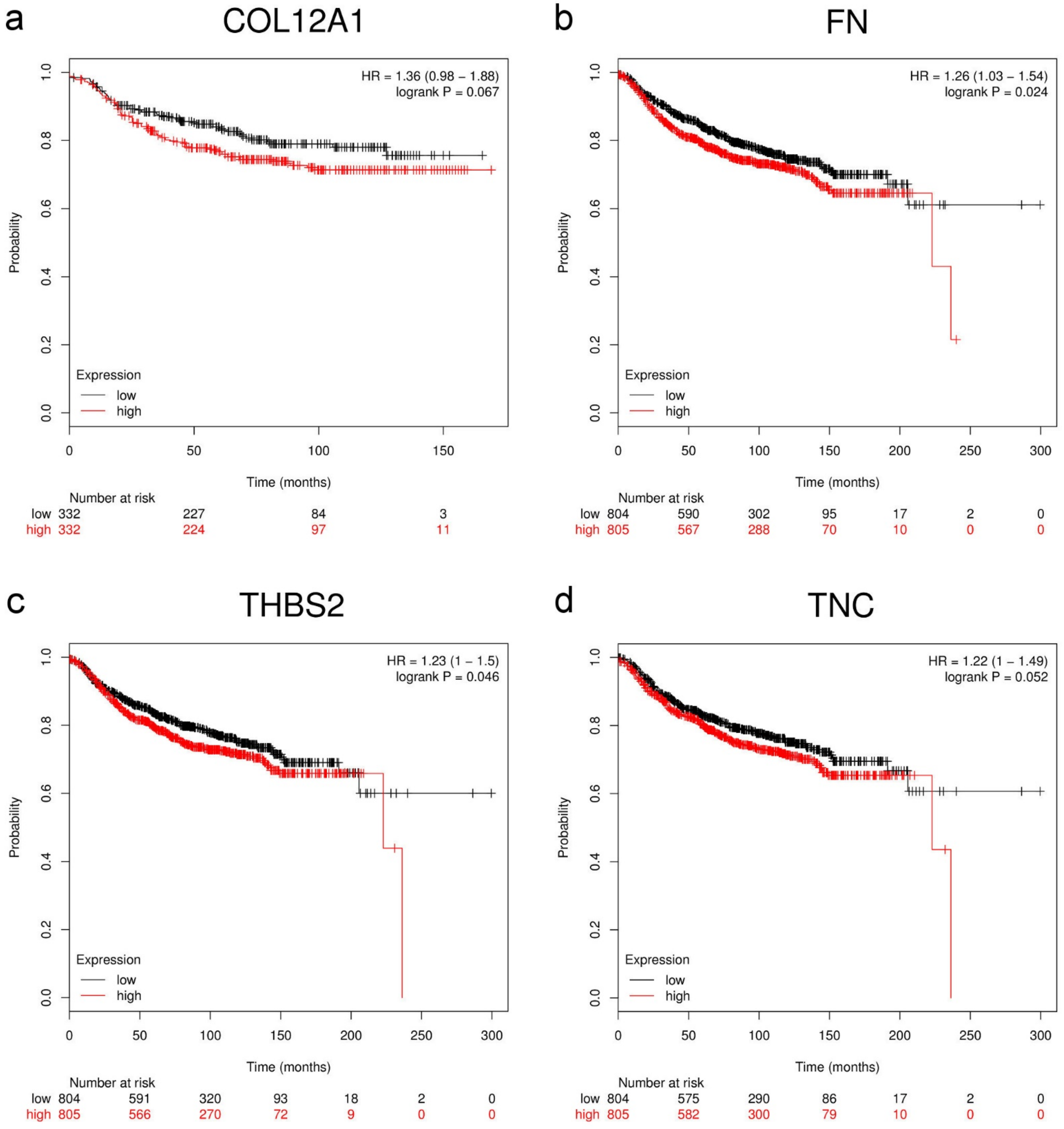

Supplementary Fig. S2. *Individual Kaplan-Meier plots of in silico distant metastasis-free survival (DMFS) for significantly decreased and increased ECM proteins.* The K-M Plotter database was used to evaluate the impact on metastasis of each protein that was significantly changed between normal and IDC tissues 38. The increased abundance proteins, (a) collagen XII, (b) fibronectin, (c) thrombospondin-2, and (d) tenascin-C, that individually correlate with, or strongly trend towards, a significant impact on metastatic outcome when highly expressed in patients. Sample size is reported under each graph, along with the hazard ratio (HR) within the graph.
